# Fertility desire and associated factors among women on the reproductive age group of Antiretroviral treatment users in Jimma Town, South West Ethiopia

**DOI:** 10.1186/s13104-019-4190-7

**Published:** 2019-03-20

**Authors:** Teshome Shiferaw, Getachew Kiros, Zewdie Birhanu, Hailay Gebreyesus, Tesfay Berhe, Mebrahtu Teweldemedhin

**Affiliations:** 1 Department of Health Education and Behavioral Sciences, Jimma University, Jimama, Ethiopia; 2 Department of Public Health, College of Health Science, Aksum University, P. O. Box: 298, Aksum, Ethiopia; 3 Department of Medical Laboratory Sciences, College of Health Science, Aksum University, Aksum, Ethiopia

**Keywords:** ART users, Women, Fertility desire, Jimma, Ethiopia

## Abstract

**Objectives:**

HIV remained the major cause of death in women of reproductive age worldwide. There is limited evidence regarding the fertility desire of HIV positive women receiving HIV care in the study area. Therefore, facility based cross-sectional study was conducted from March to April 2017 to assess fertility desire of HIV positive women and associated factors among mothers in receiving HIV care Jimma town, Southwest Ethiopia. Simple random sampling technique was taken to draw the sample after stratification. Data were analyzed using SPSS version 21 and statistical significance was declared at *P* value less than 0.05.

**Results:**

This finding showed that, 175 (46.8%) of the Antiretroviral therapy users had fertility desire with those significantly associated factors; women in the age 18–29 years [AOR = 4.05, 95% CI 1.24–13.33], being married [AOR = 0.32, 95% CI (0.13–0.78)], having diploma educational level [AOR = 5.34, 95% CI 1.10, 15.60], having only boys or girls children [AOR = 2.79, 95% CI (1.24–6.25)], having 18–36$ monthly income [AOR = 1.27, 95% CI (1.56–10.67)], Partner’s HIV status [AOR = 3.56, 95% CI (3.02–9.33)] and non use of contraceptives [AOR = 2.57, 95% CI (1.08–6.13)]. Fertility desire in the study area was high. Strengthening PMTCT service should consider fertility desire of mothers living with HIV.

**Electronic supplementary material:**

The online version of this article (10.1186/s13104-019-4190-7) contains supplementary material, which is available to authorized users.

## Introduction

HIV is one of the major causes of mortality in women of reproductive age worldwide. Sub-Saharan Africa has continued to bear the greatest burden of HIV; approximately 56% of new infections of the adult population constitute women [1]. Since the introduction of antiretroviral therapy (ART), the life expectancy of HIV-positive women has noticeably increased but challenged by many reproductive health issues. In the year 2013, around 1.5 million women living with HIV gave birth. However, a large fraction of (3.2 million) children below the age of 15 years were carriers accounting for 9.1% of all people living HIV due to vertical transmission [2–4]. Hence, programs providing highly effective treatment and prophylaxis interventions can have efficient power in reducing new HIV infections in Children [5].

The critical step towards reducing MTCT of HIV is the main pillar of the international standards for a comprehensive approach [6]. Even though PMTCT programs accounted for a 5% reduction in MTCT in developing countries, the coverage of effective ART regimens was 57% [7]. Similarly, low PMTCT coverage and poor integration with maternal services have been documented as the main challenge of the Ethiopian government [8].

In countries like Ethiopia, the complex relationship between HIV/AIDs and the high rate of fertility further threatens the preventive strategies towards the vertical spread of HIV [9]. However, Women living with HIV like the general population still prefer to give birth [10–14]. Because most HIV positive couples are in the reproductive age group, they experience difficulties in their sexual life and childbearing [13, 15].

Earlier to the PMTCT programs, health care providers have been dispiriting women living with HIV from having children in order to avoid new infections in children through many women continued to give birth with the knowledge for consequences [14–21]. Even after antiretroviral treatment, there is evidence that healthcare providers have advised women living with HIV to avoid pregnancy [21–24]. However, it is evidenced in a number of studies that many HIV positive women have children and wish more children [25–27], emphasizing the need for comprehensive care for this population who can have a safe and healthy pregnancy.

In case people living with HIV desire to have children, counseling by their health care providers has critical role to assure planned pregnancies, and minimizing prevention of mother to child transmission [28]. However, in many settings a large majority of HIV-positive women who desire more children have not discussed about reproductive health and childbearing with their health care providers [29–31]. Besides, contraception strategy has also been evidenced by the world health organization as a key strategy to reduce new infant infections [32]. In Ethiopia, studies conducted so far indicated that fertility desire in women living with HIV ranged from 34 to 45% [33–36]. However, there is still a paucity of scientific studies on the associated factors of fertility desire in the study group. Therefore, this study was aimed to assess the fertility desire and its associated factors among HIV positive women in the reproductive age group under antiretroviral treatment in Jimma town, south- west Ethiopia.

## Main text

### Methods

#### Study area and period

This investigation was done in three public health facilities at Jimma town which is 355 km far from Addis Ababa, Ethiopia to the south-west. According to the town Administrative office 2017 report, the town has an estimated 190,125 total population. The study was done from March 26 to April 30, 2017.

#### Study design and population

A facility-based cross-sectional study was conducted among women in the reproductive age group receiving ART follow-up care in the town. The study included women with HIV, aged 18 to 49 years who made at least one visit to the ART unit.

#### Sample size and sampling technique

The sample size was calculated using a single population proportion formula taking the prevalence of fertility desire among HIV-positive women’s 42.1%, margin of error 5% and adding 10% for non-response rate then finally we obtained 374 respondents. In order to have a representative respondent from the three ART units stratified sampling technique was used and the number of study subjects in each health facility was determined using Proportional allocation and individuals were taken using simple random sampling. The primary outcome variable for the study (fertility desire) was measured by answers to the question: “Are you currently planning to have (more) children in the near future?” Women’s were free to respond “Yes”, “No”, or “Don’t know, the small proportion of women who responded “Don’t Know” (if 5%) were included in the “No” category [33]. Finally, a positive (“Yes”) response to the above question was observed as fertility desire.

#### Data collection technique

Data were collected using a pre-tested structured self-administered questionnaire. The questionnaire was grouped into four categories (61 item questions or explanatory variables); socio-demographic information, HIV/AIDS disease Related factors, Knowledge of HIV transmission, Reproductive Factors and Perceived social pressure. Training was given for data collectors and supervisors on how to conduct interview and content of the questionnaire. Data was collected by four clinical nurses under two BSc. nurse supervisors and principal investigator. Daily Supervision was conducted by the supervisors and principal investigator.

#### Data exploration and method of analysis

Data was entered into EPI Data 3.1, for controlling data entry errors, and then exported to SPSS software version 21 for analysis. We use descriptive statistics and graphical techniques to explore the entire variables of the study.

Furthermore, the Bi-variant analysis was used to select best predictor variables and those variables showed significant association at a p-value of less than 0.2 were entered into multiple logistic regression models and a p-value of < 0.05 was used to see the significance in the final predictors of fertility desire. Strength and direction of the association also presented using odds ratios relative to the reference category and 95% confidence levels.

### Results

#### Socio-demographic characteristics of the study respondents

In this survey, 374 respondents were participated in making a response rate of 100%. Almost half 184 (49.2%) of the respondents who were involved in this study were in the age group of 30–39 years’ see blow (Table 1).

**Table 1:** Socio-demographic characteristics of women attending ART clinic in Jimma Town public health facilities Southwest Ethiopia, April, 2017

Socio-demographic variables	Categories	Frequency (n)	Percent (%)
Residence (n = 374)	Urban	303	81.0
	Rural	71	19.0
Marital status (n = 374)	Married	201	53.7
	Divorced/separated	91	24.3
	Widowed	55	14.7
	Never Married	27	7.2
Age category (n = 374)	18–29	161	43.0
	30–39	184	49.2
	40–49	29	7.8
Ethnicities (n = 374)	Oromo	180	48.1
	Amhara	61	16.3
	Dawuro	46	12.3
	Kefa	41	11.0
	Tigrean	24	6.4
	Others (Yem, Gurage)	22	5.9
Religion (n = 374)	Orthodox	156	41.7
	Muslim	142	38.0
	Protestant	70	18.7
	Catholic	4	1.1
	Others (heaven)	2	0.5
Occupational (n = 374)	Daily labor	82	21.9
	House wife	70	18.7
	Merchants	61	16.3
	Private employed	57	15.2
	Government employed	45	12.1
	Commercial sex worker	30	8.0
	Student	13	3.5
	Farmer	16	4.3
Educational status (n = 374)	Formal education (1–10)	122	32.6
	Unable to read and write	114	30.5
	No formal education	66	17.6
	Diploma	40	10.7
	Preparatory	25	6.7
	University degree	7	1.9
Family income (n = 374)	No income	15	4.0
	0.04–17.8$	81	21.7
	18–36$	207	55.3
	> 36$	71	19.0

#### Fertility desire of respondents

Table 2 shows that respondents who want children in the future were 175 (46.8%) and those who do not want were 199 (53.2%). Out of the 374, 172 (46.0%) discussed their Child need with health profession/counselors. From the total interviewed PLHIV’s, 50 (13.4%) want to have one or two biological live children whereas 91 (24.3%) want to have three to four children.

**Table 2:** Fertility desire among women living with HIV attending ART units at Jimma town, Southwest Ethiopia, April, 2017 (n = 374)

Variables	Categories	Frequency	Percent
Would you like to have a child in the near future?	No	199	53.2
	Yes	175	46.8
Have you discussed your fertility desire with health profession/counselor?	No	202	54.0
	Yes	172	46.0
How many children would you like to have?	None	199	53.2
	3–4 children	91	24.3
	1–2 children	50	13.4
	I don’t know	34	9.1
Now, are you using any method of contraceptive to prevent pregnancy?	Yes	200	53.5
	No	140	37.4
	Currently pregnant	34	9.1
Do know your CD4 count?	Yes	237	63.4
	No	137	36.6
Desire at least one child	Yes	93	24.9
	No	82	21.9
	Not want a child	199	53.2
Is your fertility desire related with strengthening marriage?	Yes	58	15.5
	No	117	31.3
	No, I just want a child	199	53.2
Uses ART/PMTCT to prevent transmission of the virus	Yes	72	19.3
	No	103	27.5
	Not want a child	199	53.2
Desire to replace died baby before	Yes	29	7.8
	No	146	39.0
	Not want a child	199	53.2

#### Fertility desire and associated factors

Results of the multiple logistic regression model showed that, marital status, age, educational level, monthly family income, child’s biological sex, Partner HIV status and Perceived social expectation of that partner, using contraceptive method, Parent, and community pressure to have children were found to be significantly related with fertility desire (p-value < 0.05, Table 3).

**Table 3:** Factors associated with fertility desire among HIV-positive women of reproductive age in Jimma town Southwest Ethiopia, 2017 (n = 374)

Variables	Total number	Fertility desired Number (“yes”)	COR (95% CI)	p-value	AOR
Age					
18–29	161	91 (56.5)	4.97 (1.93, 12.82)*	0.021	4.05 (1.24, 13.33)
30–39	184	78 (42.4)	2.82 (1.09, 7.25)*	0.071	2.92 (0.91, 9.35)
40–49	29	6 (20.7)	1		1
Marital status					
Never married	27	13 (48.1)	1.42 (0.59, 3.37)	0.243	2.011 (0.622, 6.501)
Married	201	112 (55.7)	1.92 (1.16, 3.18)	0.027	4.967 (1.203, 20.499)
Widowed	55	14 (25.5)	0.52 (0.916, 4.010)	0.012	0.32 (0.13, 0.78)
Divorced	91	36 (39.6)	1		1
Educational status					
Can’t read and write	27	12 (44.4)	1.27 (0.56, 2.87)	0.340	1.96 (0.49, 7.94)
Read and write but not formal	48	22 (45.8)	1.35 (0.71, 2.56)	0.935	1.054 (0.300, 3.699)
Formal education	73	42 (57.5)	2.15 (1.24, 3.74)*	0.982	1.01 (0.312, 3.21)
Preparatory	19	10 (52.6)	1.77 (1.47, 4.56)	0.672	1.38 (0.31, 6.28)
Diploma	23	18 (78.3)	5.7 (2.04, 16.12)*	0.037	5.34 (1.10, 15.60)
University degree	184	71 (38.6)	1		1
Monthly family income					
Less than 500 ETB	81	98 (63.22)	1.514 (0.492, 4.657)	0.926	1.077 (0.224, 5.188)
500–1000 ETB	207	109 (52.7)	1.27 (1.02, 7.103)*	0.001	1.79 (1.56, 10.669)
1001–5000 ETB	71	45 (63.4)	1.978 (1.106, 3.352)*	0.146	1.699 (.832, 3.468)
No income	15	7 (46.7)	1		1
Duration of ART initiation					
Less than 5 years	218	95 (43.57)	0.645 (0.348, 0.897)	0.135	0.655 (0.377, 1.141)
Greater than 5 years	156	85 (54.48)	1		1
Children’s biological sex					
All girl/s	82	42 (52.5)	1.49 (1.22, 2.609)*	0.001	1.780 (1.522, 3.852)
Both boy/s and girl/s	108	68 (65.4)	2.55 (1.285, 4.483)*	0.013	2.791 (1.246, 6.254)
No living child	88	65 (75.58)	4.18 (2.301, 7.573)	0.143	3.732 (1.667, 8.355)
All boy/s	96	40 (42.55)	1		1
Partner’s HIV status					
Positive	136	76 (55.9)	2.533 (1.585, 4.048)*	0.04	3.77 (1.03, 10.49)
Negative	73	44 (60.3)	3.03 (1.72, 5.37)*	0.005	3.56 (3.02, 9.33)
No partner	165	55 (33.3)	1		1
Partner’s interest to have children					
Yes	163	109 (66.9)	4.44 (2.86, 6.85)*	0.000	3.125 (1.811, 5.37)
No	211	66 (31.3)	1		1
Community pressure to have children					
Yes	180	104 (57.8)	2.37 (1.56, 3.703)*	0.015	1.94 (1.14, 3.31)
No	194	71 (36.6)	1		1
Use of contraceptives					
No	140	70 (50.0)	1.41 (1.212, 5.512)*	0.032	2.575 (1.082, 6.128)
Yes	200	83 (41.5)	1		1

*COR* Crude odds ratio, *AOR* adjusted odds ratio, *CI* confidence interval

*Significance for AOR

The odds of having fertility desire was 4.05 times higher among women aged 18–29 years as compared to those aged 40–49 years [AOR = 4.05, p = 0.021, 95% CI 1.24–13.33], the odds of having fertility desire was 4.9 times higher among married women as compared to those divorced [AOR = 4.967, p = 0.027, 95% CI 1.203–20.499] and the odds of having fertility desire was 0.32 times less among widowed women as compared to the divorced [AOR = 0.32, p = 0.012, 95% CI 0.13–0.78)]. The odds of having fertility desire was 5.3 times higher among diploma holder women as compared to women who completed University degree [AOR = 5.34, p = 0.037, 95% CI 1.10, 15.60]. Concerning income, the odds of having fertility desire was 1.79 times higher among those women whose monthly family income 18–35$ as compared to no income [AOR = 1.79, p = 0.001, 95% CI 1.56–10.66)]. The odds of having fertility desire was 3.7 times higher among women with HIV positive partner as compared to those without a sexual partner [AOR = 3.7, p = 0.054, 95% CI 1.03–10.49]. The odds of having fertility desire were 1.78 times higher and 2.79 times higher among mothers having children with biological sex of “all girls” and “all boys” respectively (Table 3).

### Discussion

The findings of this study show that 46.8% of the study subjects need to have children; almost comparable with the previous studies conducted in Tigray and Addis Ababa, 45.5% and 44% fertility desire respectively [37, 38] but higher as compared to the previous studies conducted in other parts of Ethiopia, 24% and 15.7% desire level respectively [39, 40]. With this regard, our figure is lower than previous findings elsewhere [41–43]. This difference might be explained by socioeconomic and cultural differences or other health-related factors including counseling skills. In this study, getting at least one child to replace themselves, strengthening a relationship, and replacing died baby were major reasons for the fertility desire of women living with HIV.

This finding indicates that partner HIV status positive is 65.0% lower than the study conducted in Nekemt Southwest Ethiopia, among women partner tested positive was 70.6% [44]. These findings show that 53.5% of the participants use any method of contraceptive; higher than the finding in Addis Ababa 43.3% [45].

In this study showed that, younger age (18–29 years), marital status (widowed and married, educational status diploma and above, having biological child sex difference both boys and girls, monthly family income (18–36$), partners of HIV positive and negative status and use of contraceptives were significant factors of fertility desire similar to previous studies conducted elsewhere [44–46].

This study indicated that participants had adequate knowledge of HIV transmission by breastfeeding 316 (84.5%) and using ART drug during pregnancy can reduce the risk of mother to children transmission 296 (79.1%); as compared to the national survey 42% of women know that HIV can be transmitted by breastfeeding and 47% of risk of vertical transmission can be minimized by using ART during pregnancy [46].

Since access to ART has improved the life expectancy of people living with HIV, ART users are willing to contemplate childbearing. In comparison to HIV free women, there was no difference in the factors that influence pregnancy decisions specifically on partner’s desire and age [47]. However, having children has great social importance in the Ethiopian context [48].

The study subjects who are unable to read and write had more (51%) desire to have children as compared to those University degrees which may be due to the fact that participants who undergone education can recognize HIV induced burden. Besides, educational level may empower women to make self-decisions on reproductive issues [49].

According to the health sector transformation plan, Ethiopia is working to create HIV free generation by the year 2030. This demands strengthening prevention of MTCT and related strategies which one of the approaches may include addressing the fertility desire of people living with HIV. Therefore, this finding will inform on policy makers and health planners working on the prevention of MTCT at local and national level. As a limitation, because the study was facility based cross-sectional study, the findings may not represent the general HIV positive women.

## Conclusion

This study revealed that almost half of the women under antiretroviral treatment had fertility desire. Being young age, married, having a biological child both sex (boys and girls), low monthly family income, diploma in educational status, none use of contraceptives, Partner’s HIV status both positive and negative, parent and community pressure were obtained as significant factors of fertility desire. Considering the fertility desire of HIV positive women, health planners and service providers should strongly focus on counseling the mothers and preventing maternal HIV transmission.

## Limitation of the study

The cross-sectional nature of the data, which makes it impossible to draw inferences about the direction of relations among study variables and the data are retrospective and thus are subject to recall bias.

## Additional file

**Additional file 1: Annex S1.** Questionnaires to assess fertility desire among ART clients in Jimma area: English Version.

## Abbreviations

AIDS: acquired immunodeficiency syndrome
ANC: Antenatal Care
ARV: antiretroviral
CD4T: 4 cells are “helper” cells
DHS: Demographic and Health Survey
MOH: Ministry of Health
HAART: Highly Active Antiretroviral Therapy
HIV: human immunodeficiency virus
NGO: Non-Governmental Organization
PLWHA: people living with HIV-AIDS
PMTCT: prevention mother to child transmission
STD: sexually transmitted disease
WHO: World Health Organization
