# Supplementary material for: Fertility desire and associated factors among women on the reproductive age group of Antiretroviral treatment users in Jimma Town, South West Ethiopia
Source: BMC Res Notes. 2019 Mar 20;12:158. doi: 10.1186/s13104-019-4190-7 (PMC6425594; doi:10.1186/s13104-019-4190-7)
Supplement: Supplementary file 1 — Additional file 1: Annex S1. Questionnaires to assess fertility desire among ART clients in Jimma area: English Version. [file 13104_2019_4190_MOESM1_ESM.docx]

# Annex S1: Questionnaires to assess fertility desire among ART clients in Jimma area: English Version

. **Part 1: Socio – demographic characteristics**

| S.No | | Question | Response | | |
| --- | --- | --- | --- | --- | --- |
| 101 | | Names of health facility | ----------------------- | | |
| 102 | | How old are you? Age(in completed year) | -------------------- | | |
| 103 | | What is your current marital status? | 1. Single 2. Married 3. Divorced 4. Widowed | | |
| 104 | | What is your Ethnicity | 1. Oromo 2. Amhara 3. Dawro 4. Kefa 5. Other, specify --------------- | | |
| 105 | | What is your educational level? | 1. unable to write and read 2. read and write only 3. Elementary (1-8 grades) 4. Secondary (9-10 grades) 5. Preparatory (11-12 grades) 6. College and above | | |
| 106 | | If you have partner what is your husband’s educational level? | 1. unable to write and read 2. read and write only 3. Elementary (1-8 grades) 4. Secondary (9-10 grades) 5. Preparatory (11-12 grades) 6. College and above | | |
| 107 | | What religion do you follow? | 1. Orthodox 2. Muslim 3. Protestant 4. Catholic 5. Other, specify ------------ | | |
| 108 | | What is your current occupation? | 1. Student 2. House wife 3. Daily laborer 4. Merchant 5. Government Employed 6. Private employed 7. Farmer 8. Commercial sex workers 9. Other, Specify ----------- | | |
| 109 | | Place of residence | 1. Rural 2. Urban | | |
| 110 | | What is your husband’s current occupation? | 1. Student 2. Daily laborer 3. Merchant 4. Government employed 5. privet employed 6. farmer 7. Other, Specify--------- | | |
| 111 | | What is your family monthly income in birr? | ------------------ | | |
| **Part II. HIV/AIDS and related characteristics** | | | | | |
| 112 | When did you have your most recent HIV test**?** (in completed year) | | | | ------------ |
| 113 | Did you voluntarily undergo the HIV test? | | | | 1. Yes 2. No |
| 114 | What was the reason for HIV testing? (Response can be as many) | | | | 1. Just I wanted to know my status 2. Referral due to suspected HIV related symptoms (e.g. TB, STI and others) 3. Spouse/partner/family member HIV positive 4. Death or Illness of spouse/partner/family member 5. Preparation for marriage or sexual relationship 6. Pregnancy related 7. Others specified |
| 115 | Have you ever disclosed your HIV status? | | | | 1. Yes 2. No If No, go to Q117 |
| 116 | If yes, Q112, to whom you were, disclosed your first HIV status? | | | | 1. Partner 2. Relative/family 3. sexual partner 4. Friend/Peers 5. Others specify---------- |
| 117 | If no, Q117, reason for non-disclosure for HIV status? | | | | 1. Fear of divorce 2. Fear of abuse 3. Fear of stigma 4. Others, specify--------- |
| 118 | Are you currently taking Anti retroviral treatment (ART)? | | | | 1. Yes 2. No If No, go to Q120 |
| 119 | When did you start ART drug? (how long since you have started the ART drug in completed year | | | | ------------------- |
| 120 | Have you ever been pregnant? | | | | 1. Yes 2. No If No, go to Q129 |
| 121 | If yes, how many times have you been pregnant? | | | | 1. ------times 2. I don’t know |
| 122 | How many live births have you had in your life? | | | | 1. -------number 2. .……I don’t know |
| 123 | How many living sons and daughters do you have? | | | | 1. Son-------------- 2. Daughter----------- |
| 124 | Have you ever given birth since you were HIV positive? | | | | 1. Yes 2. No if no go to Q128 |
| 125 | If yes, how many live births did you have? | | | | In number ---------- |
| 126 | Have you ever had a pregnancy that miscarried, was aborted, or ended in a stillbirth? | | | | 1. Yes 2. No if no go to Q129 |
| 127 | If yes, was it before or after you were HIV positive? | | | | 1. Before 2. After |
| 128 | Have you ever had a child who died at any age? | | | | 1. Yes 2. No |
| 129 | Are you pregnant now? | | | | 1. Yes 2. No if no go to Q132 |
| 130 | How many months are you pregnant now? ­­­­­­­­­­­­­­­­­­­­­­­­ | | | | 1. _____in months 2. I don’t know |
| 131 | When you got pregnant, did you want to get pregnant at that time? | | | | 1. Yes 2. No |
| 132 | Would you like to have a child? | | | | 1. Yes 2. No if no go to Q 137 |
| 133 | How many children would you like to have? | | | | 1. ____number 2. I don’t know |
| 134 | How many of these children would you like to be boys, how many would you like to be girls and for how many would the sex not matter? | | | | 1. Boys________ 2. Girls ________ 3. Either ________ |
| 135 | If yes to Q137, what is the reason for the current want to a child? | | | | 1. Want at least one child 2. To strengthen marriage 3. ART/PMTCT prevents transmission of virus 4. To replace died baby before 5. Others, specify---------- |
| 136 | Have you discussed your child needs with health profession/counselor? | | | | 1. Yes 2. No |
| 137 | Do you think your partner’s want children? | | | | 1. Yes 2. No if no go to Q 139 |
| 138 | If yes, do you think your husband/partner wants the same number of children that you want, or does he want more or fewer than you want? | | | | 1. Same number 2. More number 3. Fewer number 4. Don’t know |
| 139 | What is your husband’s/partner’s HIV status? | | | | 1. Negative 2. Positive 3. Unknown |
| 140 | If your partner was positive for HIV testing, was He starting ART? | | | | 1. Yes 2. No 3. I don’t know |
| 141 | Now, are you using any method of contraceptive to prevent pregnancy? | | | | 1. Yes 2. No |
| 142 | If question no 46 what is the reason/s for not to using family planning? | | | | 1. Fear of drug interaction 2. Trying pregnancy 3. Partner does not want contraceptive 4. Others, specify------- |
| 143 | Do you know your CD4 count (based on latest measurement)? | | | | 1. Yes 2. No |
| 144 | If yes to Q 143, what is the amount of your CD4 count? | | | | ______________ |
| **Part III. Perceived Social pressure** | | | | | |
| 145 | Does your partner want you to become pregnant? | | | 1. Yes 2. No | |
| 146 | Do you think your parents want to you to have children? | | | 1. Yes 2. No | |
| 147 | Do you think people in your community want to you to have children? | | | 1. Yes 2. No | |
| 148 | With whom you are living for the last 6 months? | | | 1. With sex partner only 2. With family 3. With peer 4. Living alone 5. Other specify------ | |
| 149 | Are you a member of any PLWHA’s association? | | | 1. Yes 2. No | |
| 150 | How long since you became of member of the association? | | | 1. ___years 2. I don’t know | |
| 151 | Have you got any support from external body due to your HIV status in the last three months? | | | 1. Yes 2. No | |
| **Part IV. Knowledge of HIV transmissions** | | | | | |
| 152 | Pregnant women, who are HIV positive, transmit the virus to their babies during the time of pregnant. | | | 1. Yes 2. No 3. I don’t know | |
| 153 | HIV can be transmitted by breastfeeding? | | | 1. Yes 2. No 3. I don’t know | |
| 154 | Using ART drug during pregnancy can reduce risk of transmission of HV from mother to children | | | 1. Yes 2. No 3. I don’t know | |
| 155 | HIV positive women who are using ART have less chances of infecting their babies during breast feeding. | | | 1. True 2. False 3. I don’t Know | |
| 156 | Women on ART have less chances of infecting their babies during delivery | | | 1. Yes 2. No 3. I don’t know | |

**Thank you for your responses and the time you spent!!**
